# Julia M. Esparza, AHIP, Medical Library Association President, 2019–2020

**DOI:** 10.5195/jmla.2019.802

**Published:** 2019-10-01

**Authors:** Jodi L. Philbrick

**Affiliations:** Senior Lecturer, Department of Information Science, College of Information, University of North Texas, Denton, TX, jodi.philbrick@unt.edu

## Abstract

In this profile of Julia M. Esparza, AHIP, Medical Library Association President, 2019–2020, she is described as a pleasure to work with, practical, and intelligent, and she adds a spark of humor to any activity. Esparza has spent the last twelve years of her career in the Health Sciences Library and Department of Medical Library Science at Louisiana State University Health Sciences Center Shreveport. She is an excellent role model for medical librarians who want to engage in scholarly activities and is active in supporting the educational needs of health care providers, students, library personnel, and the community through her instructional efforts.

With an infectious smile and a boisterous personality, Julia M. Esparza, AHIP, instantly lights up the room. One of the most amazing assets that Julia (or Julie as many know her) possesses is her knowledge of the Medical Library Association (MLA) membership. She is a walking Rolodex of members’ expertise and interests, and she enjoys sharing tailored information with members. For instance, Julie always sends information about opportunities relevant to library and information sciences students who are pursuing careers in health sciences librarianship to my colleague Ana D. Cleveland, AHIP, FMLA, regents professor, Sarah Law Kennerly Endowed Professor, and director, Health Informatics Program, College of Information, University of North Texas, and me.

I first met Julie through the South Central Chapter of MLA (SCC/MLA), and we later served on the MLA Board of Directors together. Working with Julie is always a pleasure; she is practical, is intelligent, and adds a spark of humor to any activity. She is not afraid to share her opinions on the topic of discussion. One of my fondest recollections is of her bringing a two-liter bottle of Diet Coke to a meeting to stay energized. In addition to Diet Coke and *Star Wars*, her greatest loves in life are her husband, Michael Hackett, and their son, Joseph.

**Figure:**
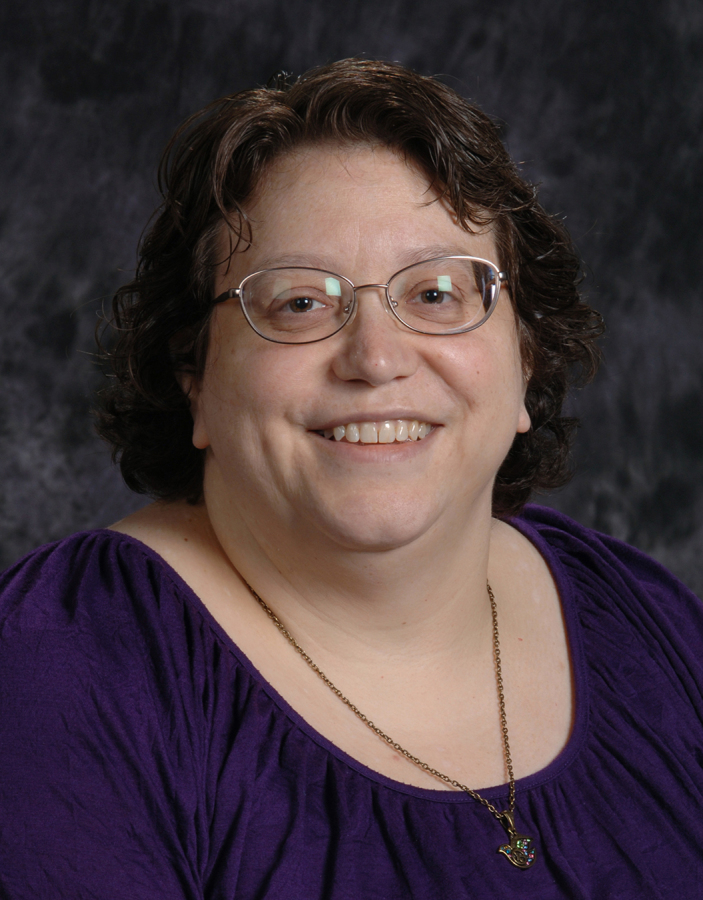


Julie has spent the last twelve years of her career in the Health Sciences Library and Department of Medical Library Science at Louisiana State University (LSU) Health Sciences Center Shreveport, where she has progressively moved up the leadership ladder and tenure-track faculty ranks from clinical medical librarian to associate director and from associate professor to professor, respectively. In 2017, she was named the Stafford and Marianne Comegys Endowed Professor in Medical Library Science.

A native of Evansville, Indiana, Julie earned her bachelor’s degree in history from the University of Evansville and completed her master’s degree in library science at the University of Indiana–Bloomington, where she also worked as a stack maintenance/public services assistant and circulation/reserve assistant at the Business/School of Public and Environmental Affairs Library.

Prior to moving to Louisiana, Julie worked as a medical librarian and manager for library services for Deaconess Health System in Evansville; electronic services librarian and serials librarian for the ECRI Institute in Plymouth Meeting, Pennsylvania; serials librarian for McNeil Consumer Products in Fort Washington, Pennsylvania; and reference/bibliographic instruction librarian for Armstrong State College (now Georgia Southern University-Armstrong Campus) in Savannah, Georgia.

Julie serves as an excellent role model for medical librarians who want to engage in scholarly activities, as she has actively published, presented, and participated in externally funded projects. Her research interests have focused on health literacy, consumer health, and outreach to clinical and consumer audiences, and she frequently collaborates with library colleagues and health care providers. She has authored or coauthored twelve peer-reviewed publications and five book chapters, with two appearing in the *Medical Library Association’s Guide to Authoritative Information Resources in the Health Sciences* and the *MLA Guide to Health Literacy*. Julie has made thirty-five refereed presentations and posters at conferences and meetings that share her knowledge, expertise, and research. She and her collaborators have frequently received research awards from MLA and SCC/MLA, demonstrating the quality of her research activities. Additionally, she has been the principal or coprincipal investigator on ten funded awards or grants.

On her Facebook profile, Julie writes that she is “a librarian who loves to teach and promote excellent patient care.” She has been active in supporting the educational needs of health care providers, students, library personnel, and the community through her instructional efforts. In addition to teaching, Julie has mentored high school, undergraduate, and medical students and collaborated with them in her research and scholarly activities. When introducing Julie for her presidential inaugural address at MLA ’19, Beverly Murphy, AHIP, FMLA, assistant director for communications and web content management, Duke University Medical Center Library, and 2018/19 MLA president, stated that Julie’s “ongoing goal is to encourage students from underrepresented groups to be sparked by the research bug and go into graduate science and health professional education.” As an example, one of Julie’s student mentees was awarded a full scholarship to attend medical school.

Julie has been a member of MLA for almost twenty years, and since joining, she has served as president-elect; member of the Board of Directors; chair of the Cancer Librarians Section, Hospital Libraries Section, and Public Health/Health Administration Section; and a member of the *Journal of the Medical Library Association* Editorial Board, in addition to her membership on numerous committees, juries, and task forces. Julie is a proud “armadillo” in SCC/MLA, and she has served the chapter through her committee work. At the state level, she served as president of the Health Sciences Library Association of Louisiana.

Not surprisingly, Julie has been honored by her MLA peers by receiving the President’s Award in 2019 for her work on the Educational Steering Committee, Lucretia W. McClure Excellence in Education Award in 2017, and the Estelle Brodman Award for Academic Medical Librarian of the Year in 2014. SCC/MLA honored her in 2018 with the Librarian of the Year Award.

Change was the major theme of Julie’s inaugural presidential address at MLA ’19, and she used a roll of pennies as an excellent metaphor for MLA members and the communities transition from sections and special interest groups to caucuses and domain hubs. As she stated, the fifty pennies in the roll can be arranged in various ways, but “no matter how these individuals are arranged, they still make up the whole.” She encouraged members to actively participate in the communities transition process in order to develop a clear vision for MLA to ensure that members’ needs are met. Her coworker, Montie’ Dobbins, AHIP, head, user access services, LSU Health Sciences Center Library Shreveport, related that Julie has been “fearless and tireless” in times of change and goes above and beyond to see that everyone is prepared to embrace change. There is no better leader than Julie to lead MLA members through the changes facing both the association and the profession.

**Jodi L. Philbrick, MSLS, PhD, AHIP**, jodi.philbrick@unt.edu, Senior Lecturer, Department of Information Science, College of Information, University of North Texas, Denton, TX

